# Altered of microRNA expression level in oligospermic patients

**Published:** 2014-10

**Authors:** Alireza Abhari, Nosratollah Zarghami, Laya Farzadi, Mohammad Nouri, Vahideh Shahnazi

**Affiliations:** 1 *Department. of Biochemistry and Clinical Laboratories, Faculty of Medicine, Tabriz University of Medical Sciences, Tabriz, Iran.*; 2 *Women’s Reproductive Health Research Center, Al-zahra Hospital, Tabriz University of Medical Sciences, Tabriz, Iran.*

**Keywords:** *mir*-*21*, *mir*-*22*, *microRNA*, *Fertility*, *Oligospermia*

## Abstract

**Background:** MicroRNA (miRNA) is small endogenous, single strand RNA molecules that regulate gene expression at post-transcriptional level through several mechanisms to affect key cellular event including male germ cells differentiation, proliferation, development and apoptosis. Mutation and/or aberrant expression of miRNAs have been associated with progression of various disorders, including infertility.

**Objective:** The purpose of this research was to study the estrogen receptor beta (ERβ(, hsa-mir-21 and, hsa-mir-22 expression level in oligospermic infertile and control fertile men and correlation between them.

**Materials and Methods: **In this study, the change in mir-21, mir-22 expression and their common target gene (ERβ) expression levels were evaluated in oligospermic infertile men (n= 43) compared with 43 age matched healthy control by Real-Time PCR methods.

**Results:** Expression analysis by qRT-PCR test on miRNA have identified that mir-21, mir-22 levels were significantly higher than those in normal controls (p<0.0001) and ERβ expression level significantly decreased in comparison with the normal group (p<0.0001).

**Conclusion:** Our study showed that mir-21 and mir-22 are indirectly involved in spermatogenesis by regulating of the estrogen receptor and might have a diagnostic and prognostic value in men infertility.

## Introduction

Spermatogenesis consists of a complex procedure of proliferation and differentiation of germ cells that leads undifferentiated diploid cells to differentiate into haploid male gamete cells. It is regulated at the transcriptional and the post-transcriptional level (1). microRNAs (miRNAs) are an essential regulator of spermatogenesis at post-transcriptional level. miRNAs are single-stranded, small (18-25 nucleotides) non-coding RNAs, which act as a regulator of genes in spermatogenesis (2).

miRNAs were first detected in human spermatozoa by Ostermeier *et al* they are abundant in spermatozoa however, the molecular features of miRNA in spermatogenesis and male fertility are not well defined (3). Understanding of these mechanisms is needed for providing high reproductive efficiency and prognostic value in fertility. Infertility affects 10-15% of couples about 50% of these cases are because of male factors (4). Oligospermia (sperm count <15×10^6^ ml) is a common cause of infertility in males, but the mechanisms that cause disease are not well known (5). 

Sperm-born mRNAs have an essential role in normal spermatogenesis. Aberrant in gene expression have a negative impact on spermatozoa differentiation and development (6, 7). Estrogen has a positive effect on sperm capacitating, fertilizing ability, as well as has a special role in male reproductive regulation (8). Cell signaling of estrogen is mediated through the estrogen receptors (ERs) that are present in the male reproductive tract, sperm and germ cells (9). It has been demonstrated that estrogen receptor gene knockout (ERKO) causes decrease in mice spermatozoa count and viability (10). Previous studies showed that mir-21 and mir-22 were predicted to target ERβ gene (11, 12). 

In this study we investigated mir-21, mir-22 and ERβ gene expression in oligospermic infertile (n= 43) compared with that of control fertile men (n= 43) to detect any difference that could provide new insights in the molecular mechanism happening during spermatogenesis.

## Materials and methods

**Study design**

The design of this study is a cross-sectional survey which was conducted in period of one year (2013). From infertile men (n=723) conferred to the Tabriz Alzahra Infertility Center (mean age 27.5±4.8 years), which despite of continuous intercourse they had a background of infertility for more than two years, 43 oligospermic infertile men were selected. Control samples (n=43), selected from normal fertile volunteers (mean age 26±3.3) had a child in the last two years and their semen analysis was normal. Neither control subjects nor patients groups did not treated with the drug for two months before sampling. The written agreement of the subjects was given according to the rules of medical ethics. This research was approved by the ethics Committees of Tabriz medical University.

**Exclusion criteria**

The volunteers with infertile partner, genital tract infection, alcohol and drug consumption, autoimmune disease, smoking and abnormality in reproductive tract were excluded from the study.

**Hormone Analysis**

The blood was placed at 37^o^C for 10 minutes to clot formation. The clot from the test tube was slightly removed then supernatant was centrifuged at 1000 × g for 10 minutes at 4^o^C. samples were analyzed for 17β-estradiol (estrogen), testosterone, follicle stimulating hormone (FSH) and luteinizing hormone (LH) using an enzyme-linked immunosorbent assay (ELISA) by commercial ELISA kit (AccuBind ELISA, Monobind, USA) according to the manufacturer's instructions.

**Isolation of spermatozoa from seminal fluid**

Semen samples were collected in a sterile flask and incubated at 37^o^C for 30 min to get the fluid then according to WHO guidelines, semen analysis was accomplished (13). Spermatozoa were purified by Goodrich methods (14). In brief, the samples were washed two times in 1× PBS buffer solution, then somatic cells were absent in SCLB solution (0.1% SDS, 0.5% TX-100 in Diethylpyrocarbonate (DEPC) water). The cells were counted, if somatic cells were, present the process was repeated. Finally the solution was frozen at -80^o^C. In this study papanicolaou staining and strict criteria were used to assess sperm morphology (15, 16).

**RNA isolation**

Total RNA was isolated using exiqon miRCURY RNA isolation kit (Exiqon, Denmark) according to the manufacturer’s instructions. Quantity and quality of the isolated RNA was measured by Nanodrop 1000 (NanoDrop ND-1000 spectrophotometer; Thermo Fisher Scientific, Waltham, MA).Total RNAs were reversed to cDNA using LNA universal RT miRNA PCR kit (Exiqon, Denmark). Briefly, 20 ng of total RNA was reverse transcribed. cDNA Synthesis was performed by thermal cycler (Eppendorf, Germany) with the following condition, value, 60 min at 42^o^C, 5 min at 95^o^C and immediately cool to 4^o^C until use. 

**Real-time PCR analysis**

Quantitative real-time reverse transcriptase-PCR was carried out by using the Corbett Rotor-Gene 6000 Real-Time PCR system (Qiagen, Germany). miRNA’s quantification was performed using MiRCURY LNA™ Universal RT microRNA PCR system (Exiqon, Denmark). Mir-16 was used as the endogenous control miRNA. ERβ relative expression was measured by qPCR with primers (ERβ: 5′-AGCCTGTTCGATCAAGTG -3′ and 5′-CCTCATCACTGTCCAGAA-3′) using SYBR Green PCR Kit (Qiagen, Germany). The expression levels were normalized to β-actin as housekeeping gene with the following primers (5′-TGGACTTCGA GCAAGAGATG-3′ and 5′-GAAGGAAGGCTG GAAGAGTG-3′). The reactions were performed in triplicate.

**Statistical analysis**

Statistical analysis was performed using SPSS software (version 18). The results were expressed as mean±SD. Genes relative expression level were calculated by using the 2^DDCq^ model (17). Unpaired Student's t-test was used to analyze the differences in gene expression between oligospermic and control group. Correlation analysis was performed using the spearman rank correlation test. In all analysis p<0.05 was considered as significant.

## Results

**mir-21, mir-22 and ERβ expression levels in oligospermic and control group**

**Figure 1:**
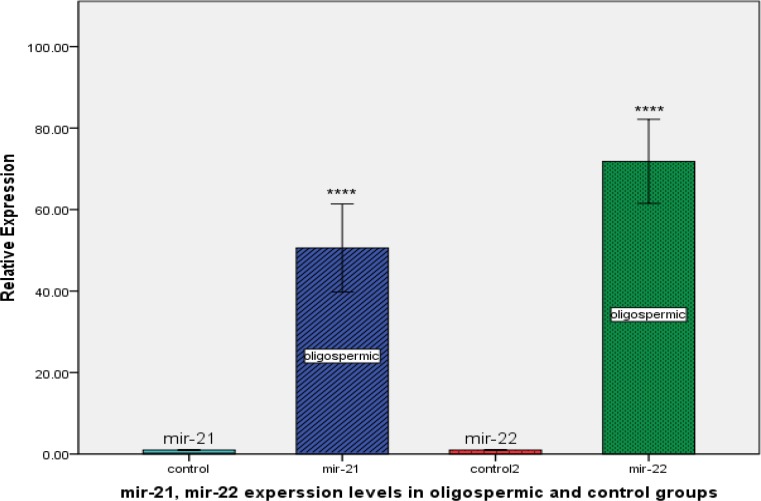
Relative expression levels of mir-21 and mir-22 in oligospermic and control group. ***P<0.0001compared with control group.

**Figure 2:**
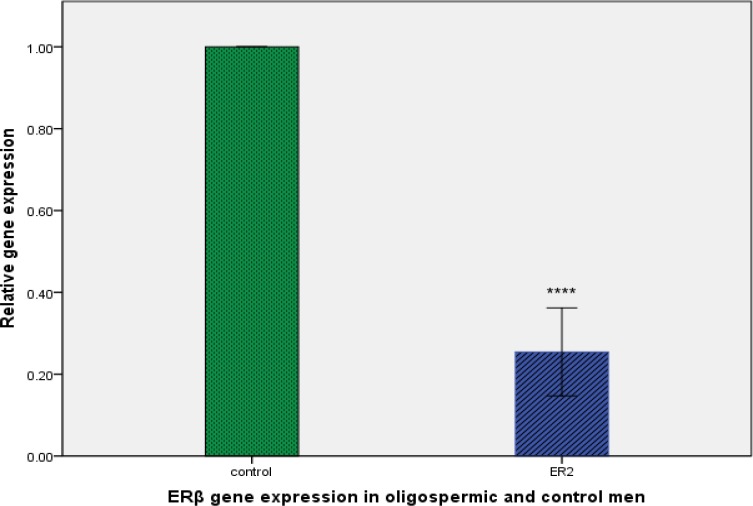
Relative expression levels of the estrogen receptor beta (ERβ) gene in control and oligospermic group. ***P<0.0001 compared with control group

We determined the expression levels of mir-21, mir-22 and ERβ in oligospermic and control group (Figure 1). By real-time quantitative RT-PCR analysis, we found that expression levels of mir-21 and mir-22 were much higher in oligospermic than control group (p<0.0001 and p<0.0001, respectively). Inversely, ERβ expression was significantly lower in oligospermic than control group (p<0.0001; Figure 2).

**Correlation between expression levels of ERβ and seminal plasma parameters**

Correlation between ERβ expression and semen were analyzed using spearman^׳^s rank correlation test (Table I). Expression levels of ERβ were strongly and positively correlated with those of sperm count, quick progressive, slow progressive and normal morphology (spearman^׳^s correlation coefficient; +0.849, +0.758, +0.753 and +0.805, respectively) and negatively correlated with immotile (spearman^’^s correlation coefficient; -0.735).

**Correlation between expression levels of mir-21 and seminal plasma parameters**

Relation between miRNAs expression and semen parameters, such as volume, sperm count, quick progressive, slow progressive, non-progressive, immotile, normal morphology and pH, was performed using Spearman^׳^s rank correlation test (Table I). Expression levels of mir-21 were negatively correlated with those of sperm count, quick progressive, slow progressive, normal morphology and pH (spearman^’^s correlation coefficient; -0.901, -0.758, -0.865, -0.906, and -0.653, respectively) and positively correlated with immotile (spearman^׳^s correlation coefficient; +0.878).

**Correlation between expression levels of mir-22 and seminal plasma parameters**

Relation between expression levels of miRNAs and semen parameters, such as volume, sperm count, quick progressive, slow progressive, non-progressive, immotile, normal morphology and pH, was performed using Spearman^׳^s rank correlation test (Table I).

Expression levels of mir-22 were negatively correlated with those of sperm count, quick progressive, slow progressive, normal morphology and pH (spearman^׳^s correlation coefficient; -0.771, -0.778, -0.779, -0.806, and -0.704, respectively) and positively correlated with immotile (spearman^׳^s correlation coefficient; +0.8) 

**Correlation between hormones and ERβ, mir-21, mir-22**

Expression levels of mir-21 were not significantly correlated with hormones but mir-22 expression levels were strongly and positively correlated with estrogen (spearman^׳^s correlation coefficient; +0.665). Expression levels of ERβ were negatively correlated with estrogen (spearman^׳^s correlation coefficient; -0.730) (Table II).

**Correlation between expression levels of mir-22, mir-21 and ERβ**

We found that expression levels of mir-21, mir-22 were strongly and negatively correlated with ERβ (spearman^׳^s correlation coefficient; -0.834, -0.0820 respectively (Table III).

**Table I:** Correlation analysis between ERβ, mir-21, mir-22 and seminal plasma parameters

**Variable**	**mir-21**	**mir-22**	**ERβ**
Volume	0.1778^a^	0.04610	-0.1449
	0.6231^b^	0.8994	0.6896
Sperm count	-0.9014*	-0.7717*	0.8495*
	0.0004	0.0089	0.0019
Quick progressive	-0.7581*	-0.7781*	0.7581*
	0.0111	0.0121	0.0111
Slow progressive	-0.8655*	-0.7796*	0.7532*
	0.0012	0.0078	0.0119
Non-progressive	-0.9131*	-0.8661*	0.7587*
	0.0002	0.0012	0.0110
Immotile	0.8781*	0.8001*	-0.7350*
	0.0008	0.0054	0.0154
Normal morphology	-0.9064*	-0.8064*	0.8057*
	0.0003	0.0004	0.0049
pH	-0.6530*	-0.7047*	0.4590
	0.0407	0.0229	0.1821

a Spearman correlation coefficient

b P, spearman’s rank correlation test

* P<0.05 is considered significant

**Table II:** Correlation analysis between ERβ, mir-21, mir-22 and hormones

**Hormones**	**mir-21**	**mir-22**	**ERβ**
FSH	0.4332^a^	0.3039	-0.3039
	0.2111^b^	0.3934	0.3934
LH	0.4720	0.2651	-0.4590
	0.1685	0.4592	0.1821
E_2_	0.4720	0.6659*	-0.7306*
	0.1685	0.0356	0.0164
Test	-0.1616	-0.2263	-0.05819
	0.6555	0.5296	0.8731

a Spearman correlation coefficient

b P, spearman’s rank correlation test

* P<0.05 is considered significant

**Table III:** Correlation analysis between mir-21, mir-22 and ERβ

	**mir-21**	**mir-22**
ERβ	-0.8345^a^	-0.8207
	0.0027^b^	0.0036

a Spearman correlation coefficient

b P, spearman’s rank correlation test

* P<0.05 is considered significant

## Discussion

Spermatogenesis is a complex procedure of male gem cells differentiation and proliferation that many genes are involved in. Disruption of the gene expression is impairs spermatogenesis and fertility. Almost mature sperm’s mRNAs are similar to those found in testis that it can show normal spermatogenesis, despite recognizing a complex population of mRNA in human mature sperm, their role and aspects affecting their expression is remain unknown. miRNAs regulate genes expression at the post- transcriptional level. They have an essential role during spermatogenesis, increase or decrease expression of miRNAs in mature sperm impair development and/or fertility (16). 

Lian *et al* showed that significant changes in miRNA expression were observed in non-abstractive azoospermic infertile patients compared with control fertile men (17). In this study we evaluated mir-21, mir-22 and their target gene (ERβ) expression levels in oligospermic infertile compared with control fertile men. We showed that mir-21 and mir-22 were significantly over expressed in oligospermic compared with the control group. We also showed that high mir-21 and mir-22 expression were associated with significant decreases in ERβ gene expression level in oligospermic group. It has been proved that mir-21 and mir-22 were predicted to target ERβ, which have been implicated in ERβ regulation (11, 12). 

Bhat-Nakshatri *et al* reported significant inverse association between the expression levels of mir-21, mir-22 and ERβ in breast cancer cells (11). Similarly, in the present study, we have showed that mir-21, mir-22 expression levels were increased unlike ERβ expression, which was decreased in sperm cells. ERβ localization in germ cell, spermatozoa, epithelial cells, Sertoli and Leydig cells which suggested the important role of ERβ in spermatogenesis (18). Lucas *et al* showed that activation of ERβ by estrogen (E_2_) increased proliferation of immature Sertoli cells (19). Sertoli cells are the somatic cells of the testis that are important for spermatogenesis. Sertoli cells accelerate differentiation of germ cells to spermatozoa, Sertoli cells dysfunction impairs spermatogenesis and fertility (20, 21). 

miRNA might be involved in spermatogenesis through other genes. Possible targets of mir-21 are MEST, AKT2, AKT1, phosphatase and tensin homolog deleted on chromosome ten (PTEN) and PLAG1 (-). PTEN and AKT1 are the targets of mir-22 (25, 26). Defect in MEST processing or action is associated with low sperm counts. MEST dysfunction was seen in idiopathic infertile men with sperm morphology below normal spermatozoa and progressive sperm motility below 40%. It can be used as a biomarker for sperm quality (27, 28). AKT1 is a serin/theronin kinas enzyme. It is the moderator of cellular growth, survival, metabolism and proliferation, in different cell types (29). 

It is proved that in Akt2-/- male mice apoptotic spermatozoa in null mice were more than wild-type mice, and sperm concentration and motility, in the null mice were significantly lower than those wild-type (30). PTEN has an important role in distinct cellular procedures, including cell survival, transformation, migration, proliferation, and moderate the germ cell differentiation (31). The pleomorphic adenoma gene 1 (Plag1) disruption in mouse affects pre- and postnatal growth and development of organs, with reproductive effects (32, 33). 

Specific miRNAs are differentially expressed in oligospermic sperm samples in comparison with normal control ejaculates. miRNAs are developing as key players in germ cell act Detailed information about miRNA expression and function will be essential to understanding the miRNA’s roles in reproductive procedures. The diverse facets of post-transcriptional regulation in male germ cells differentiation opens up new ways for contraceptive development and the survey of male fertility. Further study is required to determine the specific roles to all miRNA species involved in spermatogenesis. miRNAs could become novel targets for male contraception and for gene therapy in male infertility.

## Footnote

***This article extracted from Ph.D. thesis. (Alireza Abhari)***
